# Toward an Ultra-Wideband Hybrid Metamaterial Based Microwave Absorber

**DOI:** 10.3390/mi11100930

**Published:** 2020-10-13

**Authors:** Aicha El Assal, Hanadi Breiss, Ratiba Benzerga, Ala Sharaiha, Akil Jrad, Ali Harmouch

**Affiliations:** 1 Univ Rennes, CNRS, IETR—UMR 6164, F-35000 Rennes, France; aicha.el-assal@univ-rennes1.fr (A.E.A.); hanadi.breiss@univ-rennes1.fr (H.B.); Ala.Sharaiha@univ-rennes1.fr (A.S.); 2 Faculty of Science, LEPA- CRSI, Lebanese University, EDST, Tripoli 1300, Lebanon; akil.jrad@gmail.com (A.J.); harmush_ah@hotmail.com (A.H.)

**Keywords:** hybrid absorber, metamaterial, dielectric layer, ultra-wideband absorber, anechoic chamber

## Abstract

In this paper, we propose a novel design of an ultra-wideband hybrid microwave absorber operating in the frequency range between 2 GHz and 18 GHz. This proposed hybrid absorber is composed of two different layers that integrate a multiband metamaterial absorber and a lossy dielectric layer. The metamaterial absorber consists of a periodic pattern that is composed of an arrangement of different scales of coupled resonators and a metallic ground plane, and the dielectric layer is made of epoxy foam composite loaded with low weight percentage (0.075 wt.%) of 12 mm length carbon fibers. The numerical results show a largely expanded absorption bandwidth that ranges from 2.6 GHz to 18 GHz with incident angles between 0° and 45° and for both transverse electric and transverse magnetic waves. The measurements confirm that absorption of this hybrid based metamaterial absorber exceeds 90% within the above-mentioned frequency range and it may reach an absorption rate of 99% for certain frequency ranges. The proposed idea offers a further step in developing new electromagnetic absorbers, which will impact a broad range of applications.

## 1. Introduction

Microwave absorbing materials are becoming an important topic in most technologies and environments where the main motivation is to reduce the level of electromagnetic fields. They are especially employed in electromagnetic (EM) interference [1], radar signature [2], telecommunication [3], medical systems [4], and in EM characterization environments [5]. Many studies on microwave absorption have been performed in the frequency range 2–18 GHz [6,7,8,9], since absorbers operating in the millimeter band are highly required. For this reason, the above-mentioned frequency band will be considered in this paper. 

Traditional planar microwave absorbers, such as Salisbury screen, Dallenbach layer, and Jaumann absorber, suffer from narrow bandwidth and bulky thickness. The extensive demand of broad operating bandwidth, low profile, low density, and thin thickness absorbers have accelerated the investigation of new absorbers that meet these demands. The idea of a Metamaterial (MM) absorber was firstly proposed by Mosallaei et al. [10], and the first MM was realized by Landy et al. [11], in the gigahertz (GHz) domain. Since then, many studies have been conducted on MM for electromagnetic wave absorption in various frequency ranges, such as microwave [12,13,14,15,16], terahertz [17,18], infrared [19], and visible spectral region [20,21,22]. Additionally, today, MMs found their applications in different domains, such as radar cross section reduction [23], military target stealth missions [24], human exposure to electromagnetic fields protection [25], frequency tuning [26] telecommunication [27], and antenna gain enhancement [28].

The absorption mechanism of conventional absorbers is based on the impedance matching of lossy medium in order to obtain efficient absorbers either by changing the shape of the material (such as pyramids) to ensure a physical gradient of the impedance [29], or by stacking multiple layers with a decreased impedance, such as Jaumann layers. In this case, the EM energy is completely dissipated inside the used lossy medium [30]. 

Common electromagnetic MM absorbers consist of arrays of a conductive pattern (a periodic unit cell), a dielectric substrate, and a continuous conductive film at the back. The MMs absorption is mainly due to the local electromagnetic resonance mechanism. For this, the effective impedance of the MM structure must be matched to the impedance of free space [31]; thus, by adjusting the shape and the dimensions of the resonator, and/or the dielectric permittivity and thickness of the substrate. Consequently, at the resonant frequencies, both the transmission and reflection of the waves are minimized and absorption is maximized [31].

According to the absorption frequency range, the absorbers are classified as narrowband, broadband, wideband, single band, multi-band, or ultra-wideband absorbers. Generally, as the absorption of MM only occurs at the resonant frequency, the absorption bandwidth is considered as narrowband. Consequently, many efforts have been made in order to achieve multiple band [32,33,34,35,36,37], broadband [15,38,39,40], or ultra-wideband [6,41] microwave MM absorbers. 

The first method to broaden the absorption bandwidth of MM absorbers is to use a thick substrate. In [40], for example, a 4.2 mm thick FR-4 substrate was used to achieve an absorption higher than 90% in a wide frequency range of 4 GHz (from 4 GHz to 8 GHz), covering the entire C-band. Another proposed structure that consists of an underlined U shape resonator, with FR-4 substrate of 3.2 mm thick [38], results in a 90% absorption in the frequency band from 5.6 GHz to 9.1 GHz. 

The second method to broaden the bandwidth is based on the combination of various absorption peaks by combining multiple resonating patterns with different sizes or shapes. Consequently, multiple resonances will appear. If these frequency resonances are very close to each other, they form a broadband absorber; if not, they form a multiband absorber. For example, in [42], nine different ring resonators in the same unit cell were used. Like that, exploiting the resonators with different sizes, in order to resonate at different frequencies, results in 2 GHz absorption bandwidth (with absorption higher than 90%) between 10.5 and 12.5 GHz. Viet et al. [16] used the same idea with a dish shape resonator; a total absorption was obtained for 1.8 GHz bandwidth in the microwave region between 13.7 and 15.5 GHz. Open rings and dishes have been combined as one resonator in [43], while using different dimensions; thus, the absorption was increased (higher than 80%) for frequencies between 13.5 and 16.5 GHz. 

Another method is based on stacking multiple layers in which resonators share the same ground plane [44,45,46,47,48,49]. In this method, different resonating elements are stacked in sequence as metallic-dielectric pairs forming a two or three dimensional (two-dimensional (2D) or three-dimensional (3D)) absorbers to broaden the absorption bandwidth. In [47], three metallic films, of different dimensions of the same shape, are arranged each one between two substrates forming a 2D absorber. The resulting absorption is higher than 90% in the frequency range of 8.37–21 GHz with an absorption bandwidth of 12.63 GHz. Otherwise, other studies have stacked several layers forming a 3D MM absorber either in the form of pyramid [44] or a cylinder [45]. In the case of pyramidal shape, an absorption bandwidth of 6 GHz is achieved in the range 8–14 GHz and a dual broadband absorption bandwidth, of 2 GHz, is achieved by the cylindrical shape in the ranges of 4–6 GHz and 12–14 GHz. 

Another approach, by incorporating lumped elements into the MM resonators, was used in order to realize a broadband MM absorber [50,51,52,53,54,55,56,57]. It aims to match the input impedance with free space in a wide frequency range. In [50], for example, a Split Circle Ring (SCR) that was loaded with four lumped resistors, with R = 250 Ω, was proposed for X-band applications. MM without lumped resistors provides two resonances at 8.5 GHz and 12.5 GHz with absorptions of 26.1% and 93.9%, respectively, while the same MM structure with four lumped resistors exhibits a broadband absorption performance with absorption higher than 90% in the frequency range of 7.8–12.6 GHz [50].

Recently, it has been demonstrated that introducing MM in the design of an absorber may lead to reducing the thickness of a pyramidal absorber and enhancing its absorption performance. For example, in [58], a periodic MM structure composed of two different resonator geometries: an interleaved snake shape and spiral shape which operates in several frequency bands was proposed. The use of this proposed MM at the back of the conventional dielectric pyramidal absorber results in a compactness of this pyramidal absorber, and also an improvement of its absorption performance. This absorption was improved (up to 20 dB) between 8 GHz and 15 GHz for TE mode and between 3 GHz and 6 GHz for TM mode, with thickness being reduced by 21.7 % (total thickness of 90 mm compared to the initial thickness of 115 mm).

It should be noted here that the MM absorbers that are based on a single layer offer advantages in term of thickness, weight, and ease of fabrication. However, it is very difficult to broaden the absorption bandwidth, even if these single layer MMs associate several resonators together. As mentioned before, different methods were used to broaden the absorption bandwidth, but most of them failed to meet all of the aforementioned requirements simultaneously. In other words, the design of broadband microwave MM was mostly focused on one or two frequency bands among the C-band (4–8 GHz) [40,59], X-band (8–12 GHz) [15,50,60], Ku-band (12–18 GHz) [41], or K-band (18–27 GHz) [46], whereas only few MM designs for absorption in the S-band (2–4 GHz) [12,52] have been reported so far. For the S-band, as the EM wave has the narrowest beam width, it is considered to be an excellent candidate for radar detection. However, because of the long wavelength of the electromagnetic waves in this band, developing a high efficient microwave absorber, presenting a thin thickness, is still a big challenge. For example, Cheng et al. [52] proposed a broadband MM for the S-band while using lumped elements covering 50% of this band with an absorption higher than 90% from 3.01 to 5.28 GHz. Note that the fabrication of such absorbers is complex, which restricts their practical applications for microwave absorption.

The goal of our work is to achieve a broadband absorber with a good absorption at low frequencies, typically in the S-band. For this, first we propose a novel symmetrical MM absorber geometry, called Vshape. Secondly, in order to broaden the MM absorber bandwidth, a hybrid absorber is proposed; it consists on a combination of a thin dielectric layer to this MM to form an ultra-wideband absorber (between 2 GHz and 18 GHz).

This article is presented, as follows; first, the Materials and Methods section presents some electromagnetics notions regarding MM absorbers, the design of the novel proposed MM, the preparation of the used dielectric layer, and the characterization technics. In the second section, the properties of the dielectric layer are presented, and then, the simulation of the proposed MM and the hybrid absorber are conducted. Finally, the measurements of the realized prototypes are presented, discussed, and compared with the simulation results.

## 2. Materials and Methods 

### 2.1. Electromagnetic Absorber Theory 

A metamaterial absorber is typically a sandwiched structure that consists of an array of periodic metallic patterns, on one side of the substrate, backed with a highly conductive metallic ground plane, on the other side of the same substrate. No transmittance could be found on the other side of the MM due to the presence of the ground plane. The reflection from the MM resides at the air/MM interface; therefore, an impedance matching between the MM and the vacuum is needed in order to limit this reflection of the waves. Concretely, the effective impedance of the MM structure, as a function of the frequency (or pulsation ω) of the EM wave, is matched to the impedance of air by tuning the effective permittivity *ε*_eff_ (ω) and permeability *μ*_eff_ (ω). 

Moreover, the incident waves, which penetrated the MM, will be trapped inside it thanks to the cavity effect. The latter is formed by the space between the pattern and ground plane (the metallization at the back of the MM). Thereby, the energy of the incident waves is dissipated inside the substrate (so, inside the cavity), due to a multi-reflection (also named resonance phenomena) and the dielectric loss of this substrate. Note that the absorption frequency depends on the dielectric properties and thickness of the substrate and dimensions and the geometry of the MM. In the case of resonators with different dimensions, the incident EM waves will be trapped in several frequency bands, thus forming a multiband absorber.

For a MM absorber, as the metallic film at its back plays the role of perfect reflector and, so, blocks the transmission of the waves; the transmission T(ω) is considered to be equal to zero. Therefore, the absorption performance A(ω) can be directly calculated while using Equations (1) and (2), where S_11_ corresponds to the reflection coefficient; the lower the value of S_11_, the better the absorption performance of MM is.

(1) A(ω)=1−R(ω)

(2) $$R\left(\omega \right)={\left|S_{11}\right|}^{2}$$

### 2.2. Design of the Proposed MM 

**Figure 1:**
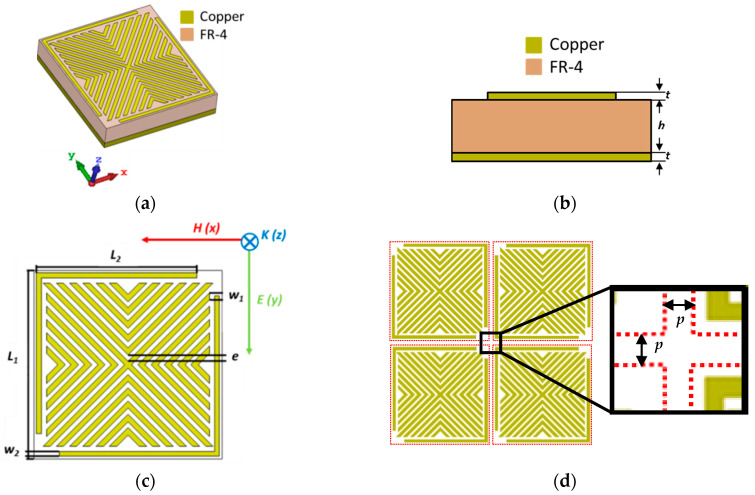
(**a**) Perspective view, (**b**) side view, (**c**) front view, and (**d**) periodic and zoom views of the Vshape Metamaterial (MM).

Figure 1 shows details of the proposed MM structure. This MM consists of three layers (Figure 1a,b). The middle layer is the FR-4 dielectric substrate with a real part of the permittivity of 4.3, a loss tangent of 0.025 and thickness *h* of 3.2 mm. The top and bottom layers are made of copper with an electric conductivity of 5.8 × 10^7^ S/m and thickness of *t* = 17 µm. The bottom copper layer is a continuous film that plays the role of a perfect reflector, while the top is formed of symmetrical copper resonators with the geometry shown in Figure 1c; it consists of 15 mm × 15 mm unit cell with a periodicity *p* = 0.04 mm (Figure 1d). Table 1 summarizes the dimensions of the Vshape resonator.

The design and the absorption performance of the proposed MM array are obtained while using (CST) Microwave Studio software. Because the unit cell is periodic in the xy-plane, Floquet port boundaries was applied in the x and y directions, whereas an open boundary is considered in the z direction. Here, the wave propagation vector k is perpendicular to the absorber plane, the magnetic field is parallel to the x-axis and the electric field is parallel to the y-axis for the TE mode, as shown in Figure 1. 

### 2.3. Elaboration of the Dielectric Layer

The dielectric layer used in this article is based on epoxy foam that was loaded with 0.075 wt.% of carbon fibers (CFs) of 12 mm length. The choice of this composite is based on our previous works showing the effect of carbon fiber length and rate [8,61] on the dielectric properties. Here, a low permittivity and moderate dielectric losses are needed.

**Figure 2:**
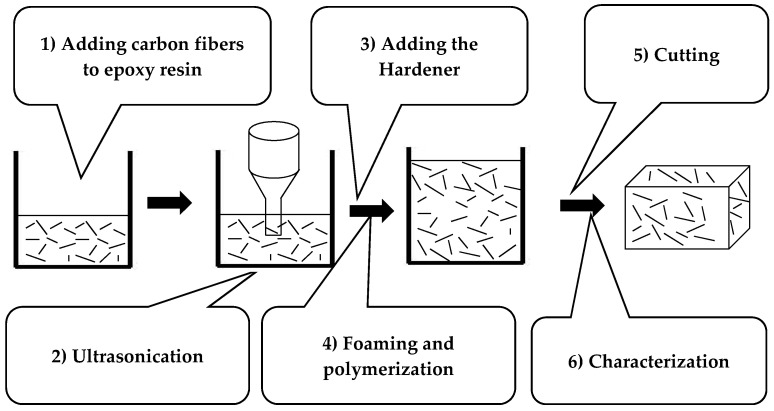
Dielectric layer elaboration steps.

Figure 2 summarizes the different steps of the elaboration method of the composite. First, the CFs (from Apply Carbon SA, Languidic, France) are dispersed in the epoxy resin (PB 170 from Sicomin, Châteauneuf-les-Martigues, France) using ultrasonication method (Sonics Materials VCX-750-220, Sonics & Materials, Inc., Newtown, CT, USA), as detailed in [61]. Subsequently, the hardener (DM 02 from Sicomin) is added with the proportions Resin: Hardener of 10:3.6. After that, the mixture is put into a mold, for foaming and polymerization steps, for six hours at room temperature.

Subsequently, the mold is placed in the oven (at 60 °C for six hours) in order to finalize the polymerization of the epoxy foam and, thus, obtaining the final rigid composite. Finally, the sample is cut to 15 cm × 15 cm × 6 cm (L × l × h), the dimensions that are needed for the dielectric characterization in the anechoic chamber. The measured density for the elaborated dielectric layer is about 0.13 g/cm^3^. Figure S1 shows SEM images and optical micrographs of the elaborated epoxy foam loaded with 0.075 wt.% of CFs.

### 2.4. Characterization Technique 

**Figure 3:**
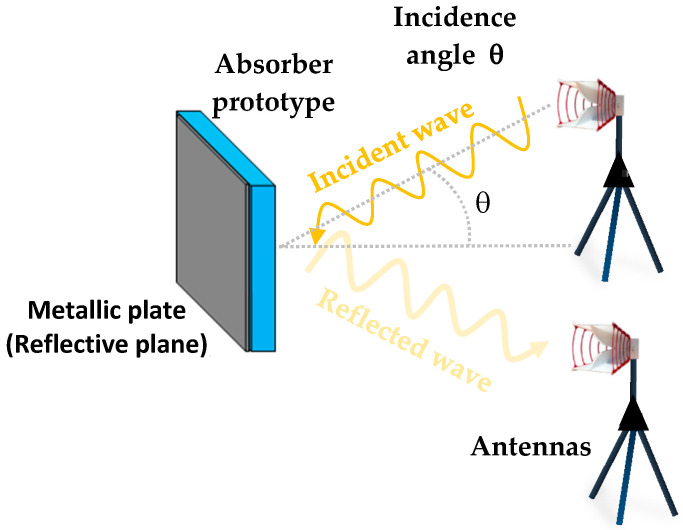
Configuration used in the anechoic chamber for the reflection coefficient measurement.

The complex permittivity of the elaborated composite has been extracted from the measurement of the reflection coefficient in the anechoic chamber, in the frequency range of 2–18 GHz. For this measurement, a quasi-monostatic configuration is used (as shown in Figure 3). For this, two horn antennas (one transmitter and one receiver) were connected to a vector network analyzer; a wave with a power of ‒8 dBm (0.15 mW) was used. 

For this technique, the measurement of four reflection coefficients is needed: on the material alone, on the material that is backed with a metallic plate, on the metallic plate alone, and on the environment of the anechoic chamber (without the material and the metallic plate). By the combination of these reflection parameters and using the Fenner et al. method [62], the extraction of the complex permittivity was done, as detailed in [63]. The same configuration was used for the measurement of the reflection coefficient of both MM and hybrid absorbers.

## 3. Results and Discussion

### 3.1. Properties of the Dielectric Layer

**Figure 4:**
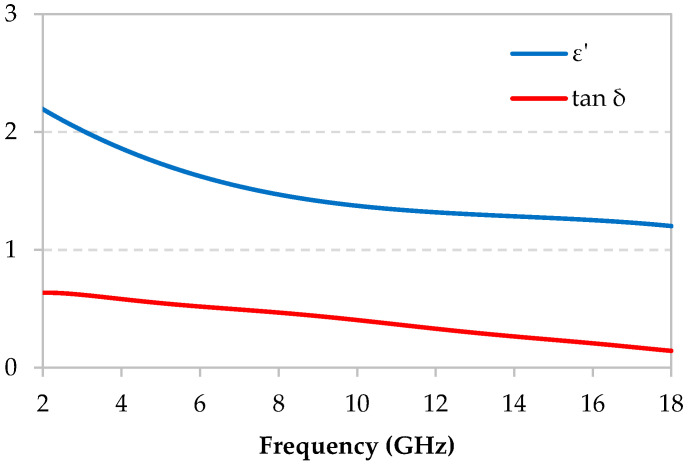
Dielectric properties of the carbon fibers (CFs) loaded epoxy foam layer (12 mm-0.075 wt.%).

The composite that was based on epoxy foam loaded with 0.075 wt.% of 12mm-length CF was prepared and measured in the anechoic chamber. Figure 4 shows the extracted dielectric properties. The real part of the permittivity *ε*’ and the dielectric losses tan*δ* both decrease with respect to frequency. Indeed, these properties decrease from the values of *ε*’ = 2.20 associated with tan*δ* = 0.64 (at 2 GHz) to the values of *ε*’ = 1.20 associated with tan*δ* = 0.15 (at 18 GHz). Figure S2 shows dielectric properties of composites loaded with various weight percentages of CFs. These figures show, as expected, an increase of the properties as a function of the CFs rate.

### 3.2. EM Wave Absorption Performance: Simulations

**Figure 5:**
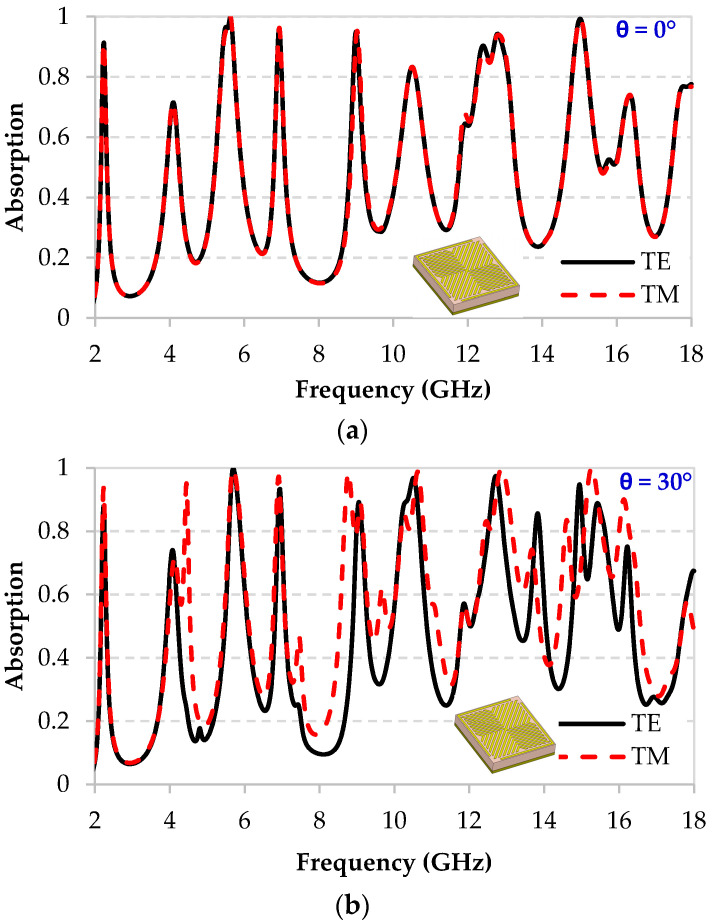
Simulated absorption performance of the MM for TE and TM modes at (**a**) normal incidence θ = 0° and (**b**) oblique incidence of θ = 30°.

The proposed MM structure has been studied under normal and oblique incidences for TE and TM polarizations; Figure S3 illustrates the detailed setup of these configurations. The simulated absorption performance of the proposed multiband MM at normal incidence (θ = 0°) for both TE and TM polarizations is illustrated in Figure 5a. The proposed MM exhibits several distinct absorption peaks, the highest among them (with absorption > 90%) occurs at 2.2 GHz, 5.63 GHz, 6.94 GHz, 8.99 GHz, 12.8 GHz, and 15.01 GHz, corresponding to an absorption of 91.29%, 99.9%, 96.09%, 94.96%, 94.2%, and 99.2%, respectively. Furthermore, the absorption peaks and intensity of the proposed MM are unchanged for both TE and TM polarizations, as expected, due to its symmetrical shape. 

Figure 5b illustrates the absorption performance of the proposed multiband MM at oblique incidence of θ = 30° for both TE and TM polarizations. It exhibits several distinct absorption peaks that differ between TE and TM. For the TE mode, the highest peaks (with absorption > 90%) occur at 5.7 GHz, 6.9 GHz, 10.5 GHz, 12.7 GHz, and 14.92 GHz, corresponding to an absorption of 99.7%, 93.31%, 96.74%, 97.39%, and 94.74%, respectively. For TM mode, the highest peaks (with absorption > 90%) occur at 5.6 GHz, 6.9 GHz, 8.7 GHz, 10.6 GHz, 12.8 GHz, and 15.27 GHz, corresponding to an absorption of 99.4%, 97.29%, 98.4%, 98.81%, 99.64%, and 99.96%, respectively.

It can be noticed that the absorption rate is almost similar between the TE and TM modes when dealing with normal incidence. However, when it comes to oblique incidence a clear difference, in the absorption rate, between the TE and TM polarizations, is observed (see Figure 5). In brief, the TE and TM results of any symmetric structure will be the same for normal incidence and different for oblique incidences. 

Figure S4 presents a comparison of the absorption resonances between normal and oblique incidences. Almost all of the obtained peaks for both angles of incidence are the same, but with two new resonances that appeared for the oblique incidence of 30° at 13.8 GHz and 15.4 GHz with 85.6% and 88.8% absorption rates, respectively. 

**Figure 6:**
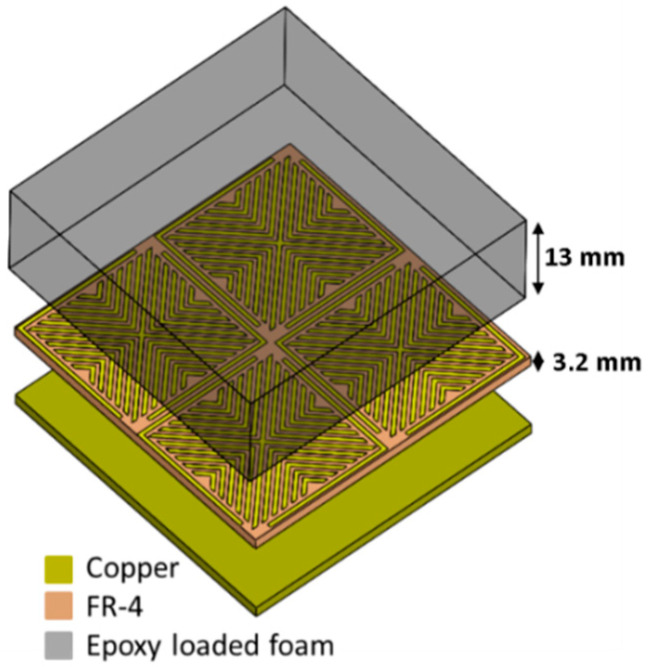
The proposed hybrid absorber.

The dielectric composite based on CF loaded epoxy foam is used to broaden the absorption bandwidth of the proposed MM. The advantage of this dielectric layer lies in both the used low load of the CFs and the used thin thickness of this composite to form the hybrid absorber. Figure 6 shows the proposed structure of the ultra-wideband absorber. It is composed of the proposed MM of 3.2 mm thickness and of the epoxy foam composite (loaded with 12 mm CF of 0.075 wt.%), with 13 mm thickness, mounted on the top of the MM. The total thickness of the proposed planar hybrid absorber is of 16.2 mm. 

It should be noted here that, for this hybrid material, EM absorption will be provided not only by the MM structure, but also by the composite layer. Indeed, the latter ensures, on the one hand, an impedance matching at air/absorber interface (thanks to its low permittivity) that allows for a smooth penetration of the EM waves in the absorber and, on the other hand, an absorption of the EM waves thanks to its moderate dielectric losses.

Before presenting the simulation of the absorption performance of the chosen hybrid structure (with a composite loaded with 0.075 wt.% of CFs), Figure S5 shows the simulation of the reflection coefficient of the hybrid absorber that was carried out using the dielectric properties of different composites with different CFs loads (dielectric properties shown in Figure S2). These simulations are compared to the one of the MM absorber alone. Figure S5 shows that a compromise must be made in order to obtain the best absorption performance and the largest bandwidth. Indeed, Figure S5a shows that, when lower CF rates are used (0.025 wt.% or 0.05 wt.%), the reflection of the MM is improved, but the losses are too low to broaden the bandwidth, and an uninterrupted large bandwidth (Γ < ‒10 dB) could not be obtained. Contrary to that, when higher CFs loads are used (0.1 wt.% or 0.2 wt.%), the complex permittivity is higher. This high permittivity no longer ensures the impedance matching and, so, induces a higher reflection (Figure S5b) at the air/hybrid absorber interface (especially in the frequency range between 3 GHz and 8 GHz), which affect the absorption performance. These simulations confirm that, for the hybrid absorber, a low *ε*’ that is near to that of air (ensuring an impedance matching at the air/material interface and so, the lowest reflection) and moderate dielectric losses (that ameliorate the absorption performance) are needed.

**Figure 7:**
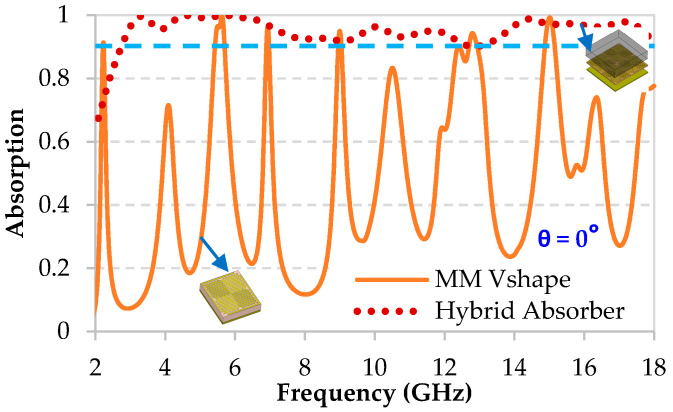
Simulation results of the absorption performance of the hybrid absorber compared to that of the MM for the normal incidence θ = 0° (the blue dashed line corresponds to the absorption value of 90%).

Figure 7 shows the simulated absorption performance, at normal incidence for both TE and TM polarizations, of the chosen hybrid absorber (using the 0.075 wt.% CFs loaded composite). An important absorption rate higher than 90% is obtained in an ultra-wide frequency range from 2.6 GHz to 18 GHz, covering 70% of the S-band and the entire C-band, X-band, and Ku-band.

Figure S6 shows the simulated absorption performance at oblique incidence of 30° for both TE and TM polarizations of the proposed hybrid absorber. For both TE and TM polarizations, a significant absorption rate higher than 90% is obtained in an ultra-wide frequency range from 2.6 GHz to 18 GHz, covering 70% of the S-band and the entire C-band, X-band, and Ku-band. A better absorption performance for the TM polarization, as compared to the TE polarization, is expected (Figure S6). 

### 3.3. EM Wave Absorption Performance: Measurements 

**Figure 8:**
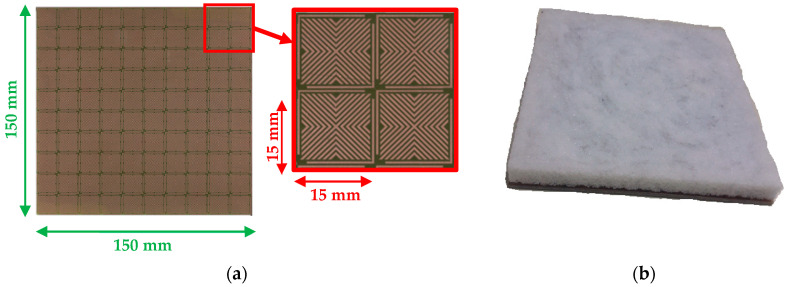
(**a**) Elaborated Vshape MM and (**b**) the final hybrid absorber.

The proposed MM absorber is fabricated with 10 × 10 unit cells with a total dimension of 150 mm × 150 mm × 3.2 mm while using laser printing technique with LPKF laser machine of the IETR laboratory. Figure 8a shows the top view of the fabricated structure. The elaborated dielectric layer that is made of epoxy loaded foam is cut to the desired thickness, 13 mm. This latter is glued on the top layer of the elaborated MM to compose the proposed hybrid absorber (Figure 8b).

**Figure 9:**
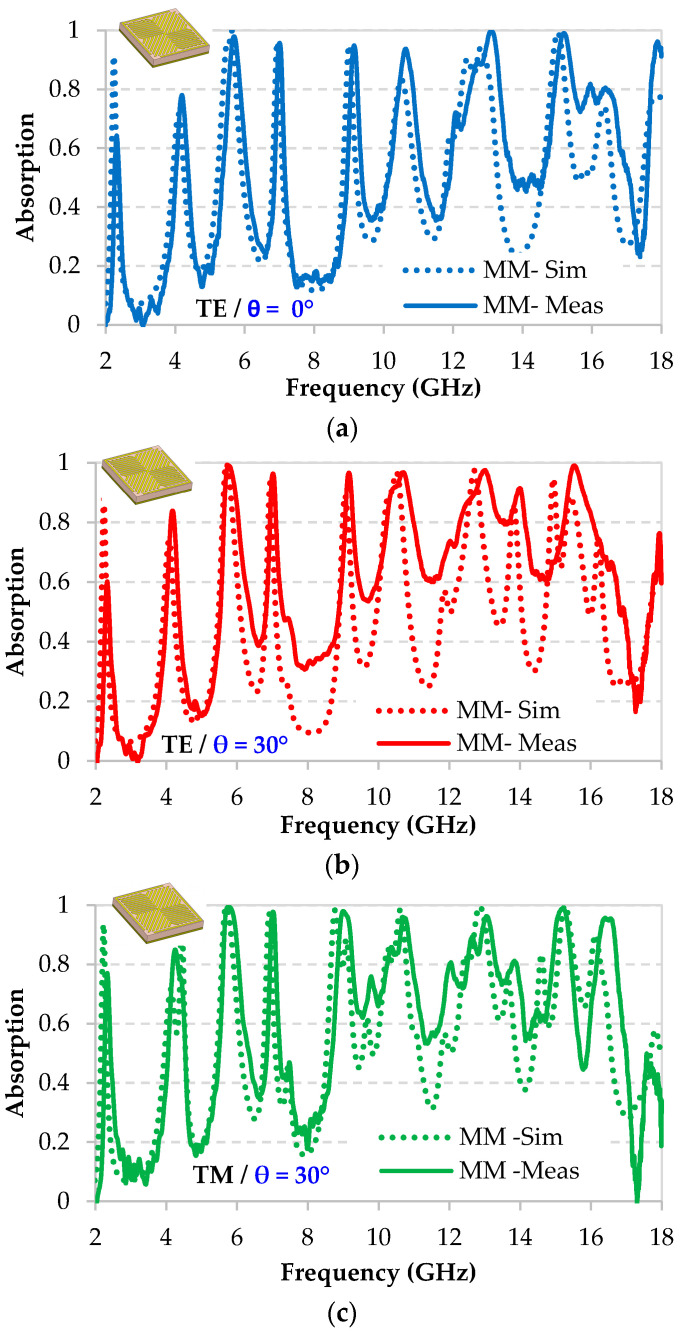
Comparison between measured and simulated absorption performance of the proposed MM at (**a**) normal incidence θ = 0° and (**b**) for TE and (**c**) TM modes at oblique incidence of θ = 30°.

From the measured reflection coefficient, in Figure 9 we present the deduced absorption performance for TE and TM polarizations at normal incidence (Figure 9a) and at oblique incidence of 30° (Figure 9b,c). These measurements are compared to the simulation results. It can be noted here that good agreement between measurements and simulations is obtained and the same resonances are observed, even if the absorption level is slightly different. For example, for the normal incidence (Figure 9a), a lower intensity is obtained at 2.3 GHz, with an expected absorption of 90% by simulation and an obtained absorption of 63% by measurement. For the other resonances, the same or even better absorption is obtained; for example, at 4.2 GHz, the expected absorption by simulation is 70.9% while an absorption of 77% is achieved by measurement. The weak difference observed between the simulation and measurement results is probably due to a geometric deviation between the perfect simulated structure of the MM and the produced one.

Likewise, the agreement between simulation and measurement can be noted for both TE and TM modes at an oblique incidence of 30°. For TE (Figure 9b), the same resonances are observed with a lower intensity only at 2.3 GHz, with an expected absorption of 93% by simulation and an obtained absorption of 76% by measurement. However, the same or even better absorption for the other resonances is obtained. For TM (Figure 9c), the same resonances are also observed with a lower intensity at 2.3 GHz, with an expected absorption of 93% by simulation and an obtained absorption of 76.9 % by measurement; the same or even better absorption performance for the other resonances is confirmed. However, the most obtained resonances have absorption rate higher than 90% as predicted with simulation. This MM is considered as a good multiband absorber, despite the slight difference between simulation and measurement results.

**Figure 10:**
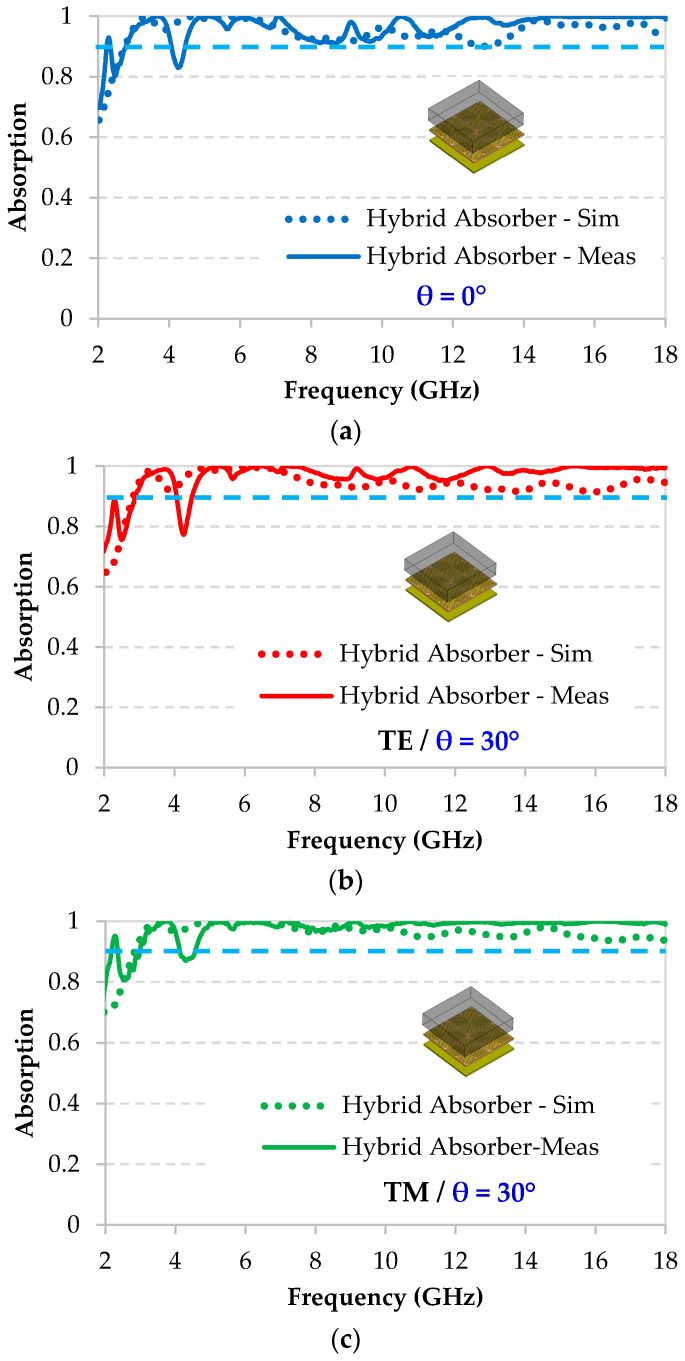
Comparison between simulated and measured absorption performance, of the hybrid absorber at (**a**) normal incidence θ = 0° and (**b**) TE and (**c**) TM modes for oblique incidence of θ = 30°.

The measured absorption performance of the hybrid absorber at normal incidence, as well as, at an oblique incidence of 30° for TE and TM polarizations are presented in Figure 10a‒c, respectively. These measurements are also compared to the simulation results. Note that, a good agreement between measurements and simulations is obtained and it confirms a high absorption performance greater than 90% for frequencies between 2.6 GHz and 18 GHz, excluding the frequency range between 4.10 GHz and 4.45 GHz. The decrease of the absorption level in this narrowband is due to the relatively high coupling, in this frequency band, and between the horn antennas used for the measurements in the anechoic chamber.

Otherwise, a better performance is obtained through measurements for the entire studied frequency band. For example, at 13 GHz, an absorption of 99.5% is obtained by measurement, while the predicted absorption by simulation is 90% at normal incidence. At oblique incidence of 30°, an absorption rate of 99.9% is obtained by measurement while the predicted absorption by simulation is 92.2% for TE polarization. The uncertainty on the dielectric properties, as extracted from the measurement in anechoic chamber and used for the absorption simulation, may be at the origin of this slight observed difference between simulation and measurement results.

Simulations of the absorption performance for other incidence angles were conducted, and these simulations are shown in Figure S7; the measurement for these angles of incidence was not carried out because of the space restrictions in the anechoic chamber used. 

Figure S7 presents the simulation results for TE (Figure S7a) and TM (Figure S7b) polarizations for θ varying between 0° and 60°; Figure S7c and Figure S7d show the same simulations for only incidence angles of 0°, 15°, 30°, 45°, and 60°, for TE and TM polarizations, respectively. These simulations predict an absorption performance that is higher than 90% between 2.6 GHz and 18 GHz, for the different incidence angles. However, for the TE mode and highest angles (θ ≥ 45°), the absorption performance is slightly affected, in low (<4.5 GHz) and high (>16 GHz) frequencies (Figure S7c). 

Finally, the absorption performance of the proposed hybrid absorber is compared with the other reported structures of microwave MM absorbers. Table 2 presents the MM characteristics in terms of the frequency range with an absorption higher than 90%, absorption bandwidth, covered bandwidth, and the method of broadening bandwidth. It can be observed that the proposed structure has an absorption higher than 90%, between 2.6 GHz and 18 GHz, forming an ultra-wideband hybrid absorber that covers 70 % of the S-band (2.6–4 GHz) and the entire C-band (4–8 GHz), X-band (8–12 GHz), and Ku-band (12–18 GHz); whereas, other achieved MMs only cover one or two bands. As a conclusion, the hypothesis of broadening the absorption bandwidth of the proposed multiband MM by associating a thin, light, and slightly CFs loaded dielectric layer is confirmed.

## 4. Conclusions

In this work, a new geometry of multiband MM absorber is proposed. A thin, light, and slightly charged dielectric layer, made of 0.075 wt.% CFs loaded epoxy foam, is associated to the front of this proposed MM absorber, in order to broaden the absorption performance. The choice of this composite is based on the necessary compromise between a low permittivity (to ensure the impedance matching at the air/absorber interface) and a moderate dielectric loss in order to improve the absorption performance. The dielectric layer has a thickness of 13 mm and the proposed MM has a thickness of 3.2 mm. The hybrid absorber presents a total thickness of 16.2 mm with an absorption performance that is greater than 90% from 2.6 GHz to 18 GHz, for TE and TM polarizations of normal (θ = 0°) and oblique (θ = 30°) incidences. As a result, an ultra-wideband, light, and compact absorber that covers 70 % of the S-band (2.6–4 GHz) and the entire C-band (4–8 GHz), X-band (8–12 GHz), and Ku-band (12–18 GHz) is achieved. A good agreement between measurement and simulation is obtained for both normal (θ = 0°) and oblique incidence (θ = 30°) for TE and TM modes, which validates this proposed hybrid absorber.

## Figures and Tables

**Table 1:** Parameters of the proposed MM.

Parameters	Value (in mm)
*t*	0.017
*h*	3.2
*L_2_*	12.8
*L_1_*	15
*w_1_*	0.6
*e*	0.5
*w_2_*	0.4
*p*	0.04

**Table 2:** Comparison between the proposed structure of this work and other presented MMs of literature.

Reference	Absorption > 90%	Absorption Bandwidth	Covered BW	Method of Broadening the BW
[52]	3.01–5.28 GHz	2.27 GHz	50% of the S-band	incorporating lumped elements
[38]	5.6–9.1 GHz	3.5 GHz	87.5% of the C-band	Thick substrate
[40]	4–8 GHz	4 GHz	C-band	Thick substrate
[16]	13.7–15.5 GHz	1.8 GHz	30% of the Ku-band	Combining multiple resonating structures
[42]	10.5–12.5 GHz	2 GHz	50% of the X-band	Combining multiple resonating structures
[43]	13.5–16.5 GHz (>80%)	3 GHz	50% of the Ku-band	Combining multiple resonating structures
[44]	8–14 GHz	6 GHz	X-band and 33.3% of the Ku-band	stacking multiple layers in 3 D
[45]	4–6 GHz and 12–14 GHz	2 GHz for each BW	50% of the C-band and 33.3% of the Ku-band	stacking multiple layers in 3 D
[47]	8.37–21 GHz	12.63 GHz	X-band And Ku-band	stacking multiple layers in 2 D
[50]	7.8–12.6 GHz	4.8 GHz	X-band	incorporating lumped elements
This work	2.6–18 GHz	15.4 GHz	70 % of the S-band Entire of C-band, X-band and Ku-band	Associating a thin dielectric layer to the MM

## Supplementary Materials

The following are available online at https://www.mdpi.com/2072-666X/11/10/930/s1, Figure S1: SEM images of the epoxy foam loaded with 0.075 wt.% of CFs, Figure S2: Real part of the permittivity and dielectric losses of epoxy foams loaded with different CFs weight percentages, Figure S3: Setup under TE and TM polarizations, Figure S4: Simulated absorption performance of the MM, Figure S5: Simulation of the reflection coefficient of the MM alone and the hybrid absorber based on composites loaded with CFs loads ≤ 0.075 wt% and CFs loads ≥ 0.075 wt%., Figure S6: Simulated absorption performance of the hybrid absorber compared to that of the MM at oblique incidence of θ =30° for the TE and for the TM polarizations, Figure S7: Simulation of the absorption performance of the hybrid absorber with different incidence angles.
