# Supplementary material for: Toward an Ultra-Wideband Hybrid Metamaterial Based Microwave Absorber
Source: Micromachines (Basel). 2020 Oct 13;11(10):930. doi: 10.3390/mi11100930 (PMC7601967; doi:10.3390/mi11100930)
Supplement: Supplementary file 1 [file micromachines-11-00930-s001.pdf]

micromachines

Supplementary Materials: Toward an Ultra-Wideband Hybrid Metamaterial Based Microwave Absorber

Aicha El Assal, Hanadi Breiss, Ratiba Benzerga, Ala Sharaiha, Akil Jrad and Ali Harmouch

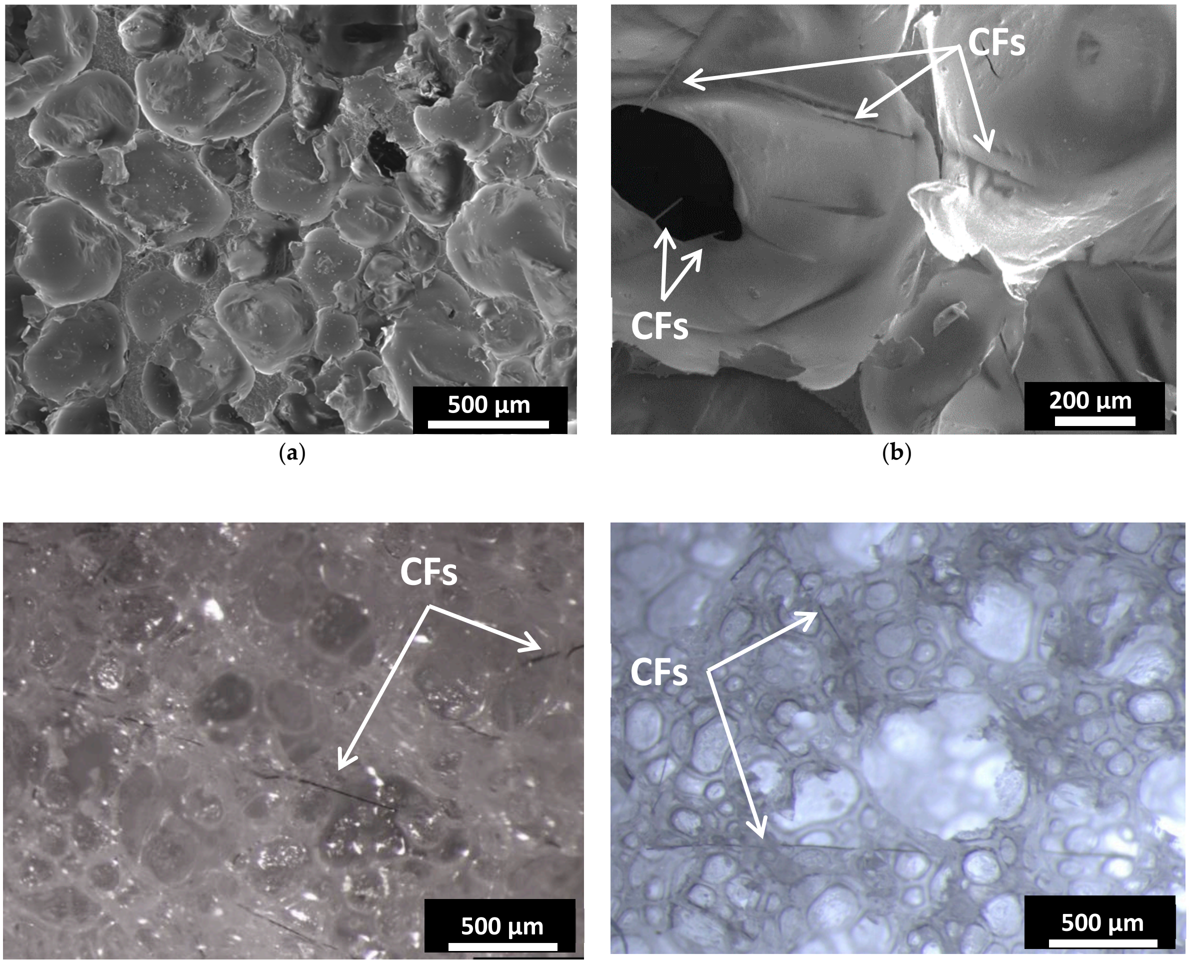

(**a**) (**b**)

(**c**) (**d**)

Figure S1. SEM images of the epoxy foam loaded with 0.075 wt.% of CFs with (**a**) a global view of the composite and (**b**) a zoom view on CFs embedded in the composite; Optical micrographs of the epoxy foam loaded with 0.075 wt.% of CFs obtained by (**c**) a reflection and (**d**) a transmission modes of the microscope.

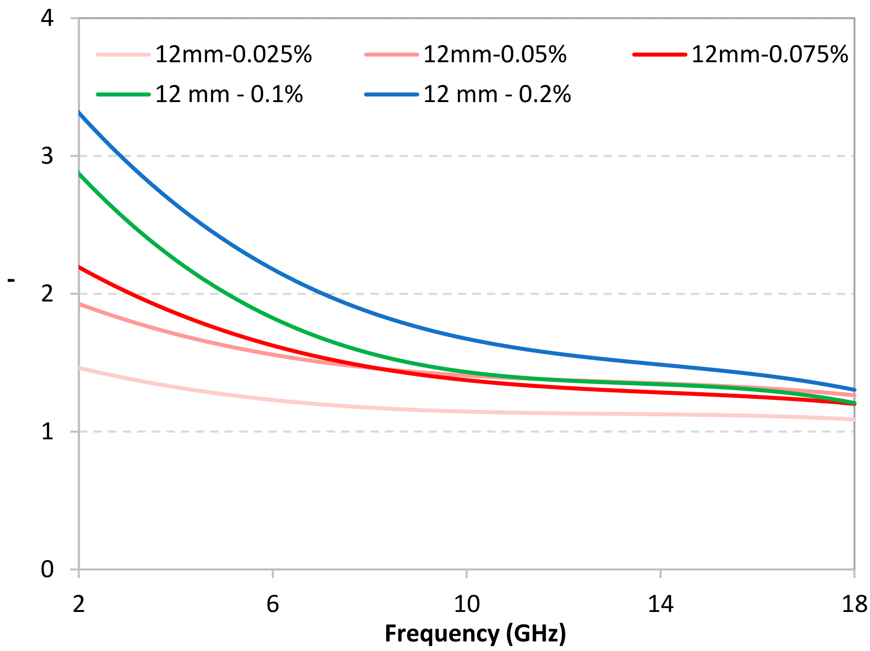

(**a**)

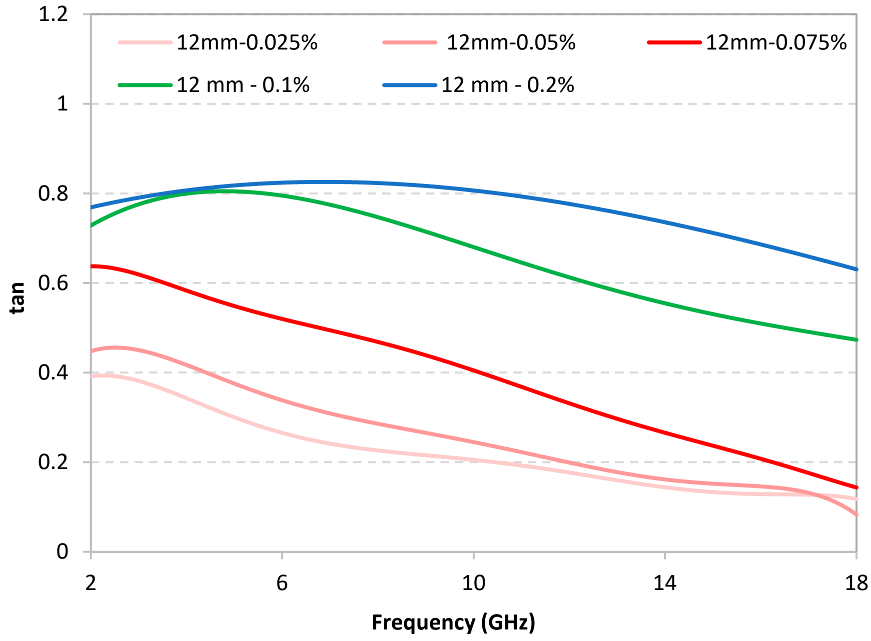

(**b**)

Figure S2. (**a**) Real part of the permittivity and (**b**) dielectric losses of epoxy foams loaded with different CFs weight percentages.

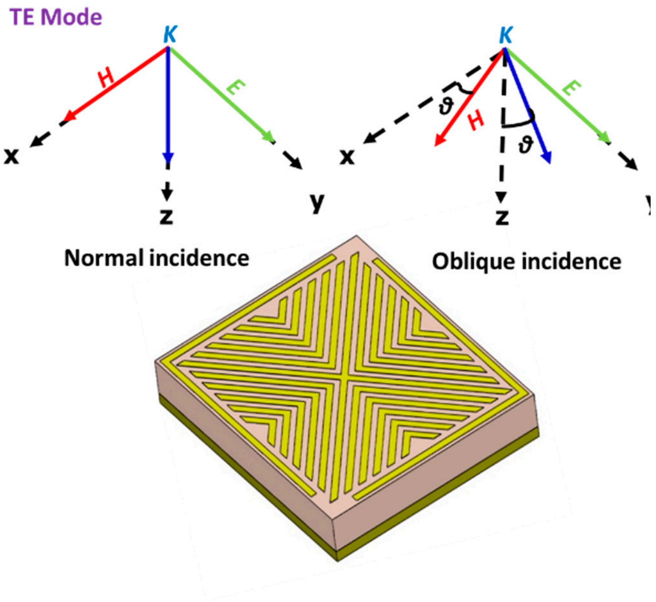

(**a**)

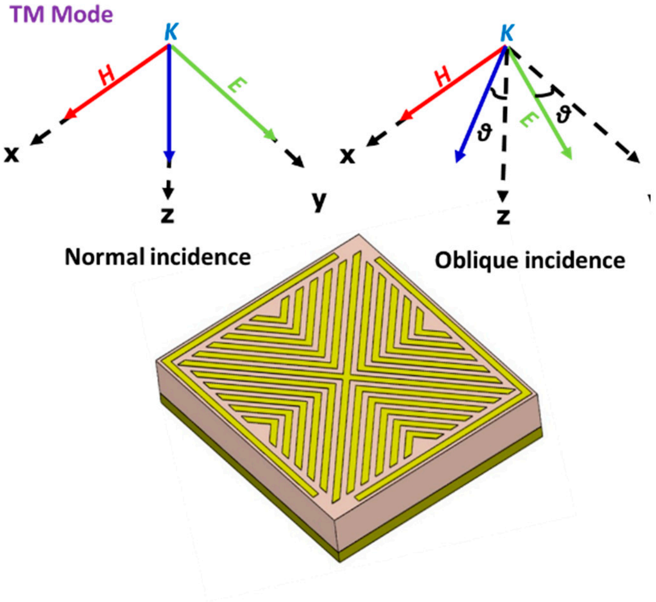

(**b**)

Figure S3. Setup under (**a**) TE and (**b**) TM polarizations.

Under normal incidence (θ = 0°), and for both TE and TM polarizations, the electric field is always aligned towards y-axis, the magnetic field is aligned towards x-axis and the wave propagation is always aligned towards the z-axis.

Under oblique incidence(θ ≠ 0°), and for TE, the electric field is aligned towards y-axis whereas the directions of magnetic field and wave propagation are varied simultaneously by an angle θ with respect to x and z directions, respectively. Under oblique incidence, and for TM, the magnetic field is always aligned towards x-axis whereas the directions of electric field and wave propagation are varied simultaneously by an angle θ with respect to y and z directions, respectively.

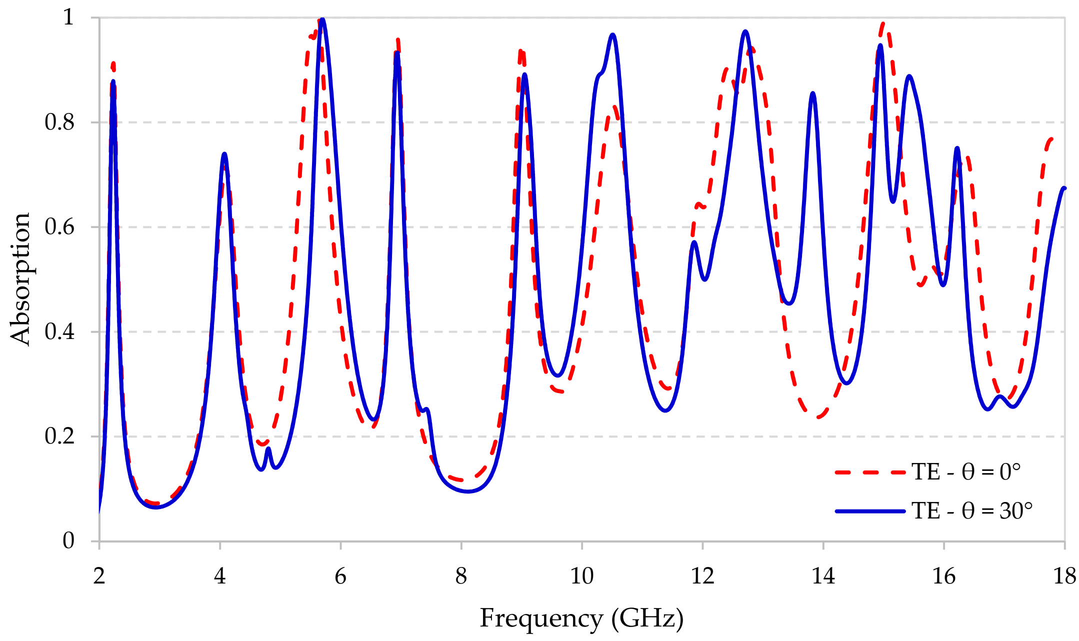

(**a**)

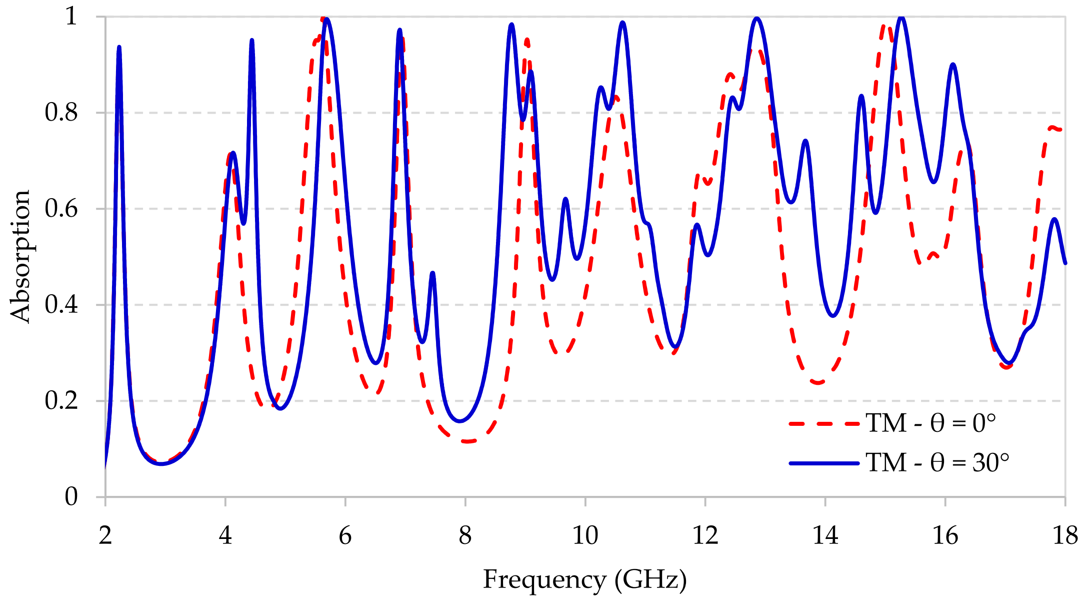

(**b**)

Figure S4. Simulated absorption performance of the MM for (**a**) TE and (**b**) TM modes at normal θ = 0°and oblique incidence of θ = 30°.

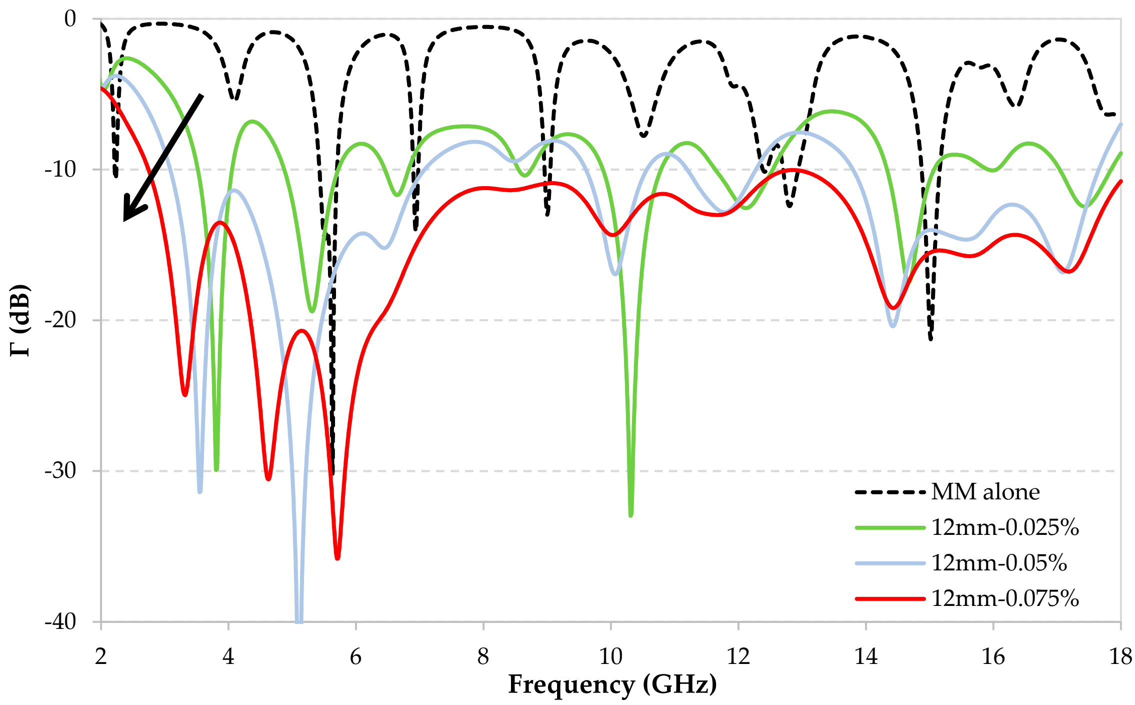

(**a**)

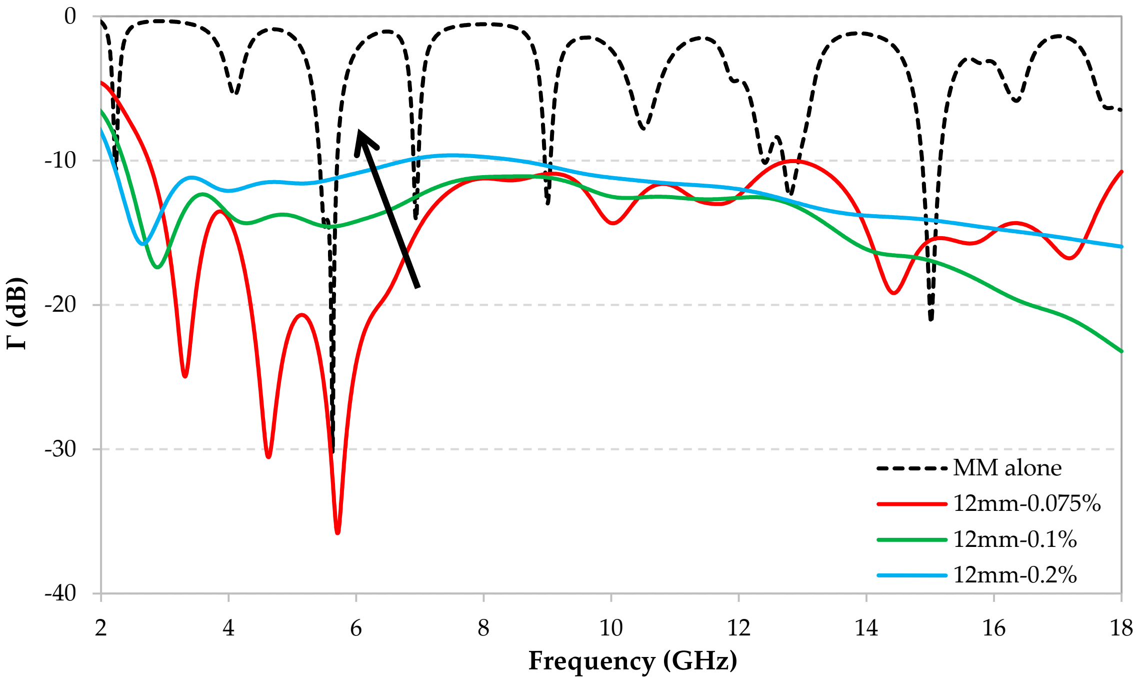

(**b**)

Figure S5. Simulation of the reflection coefficient of the MM alone and the hybrid absorber based on composites loaded with (**a**) CFs loads ≤ 0.075 wt% and (**b**) CFs loads ≥ 0.075 wt%. The black arrow shows the increase of the CFs rate. Simulations were done for a normal incidence of EM waves (θ = 0°).

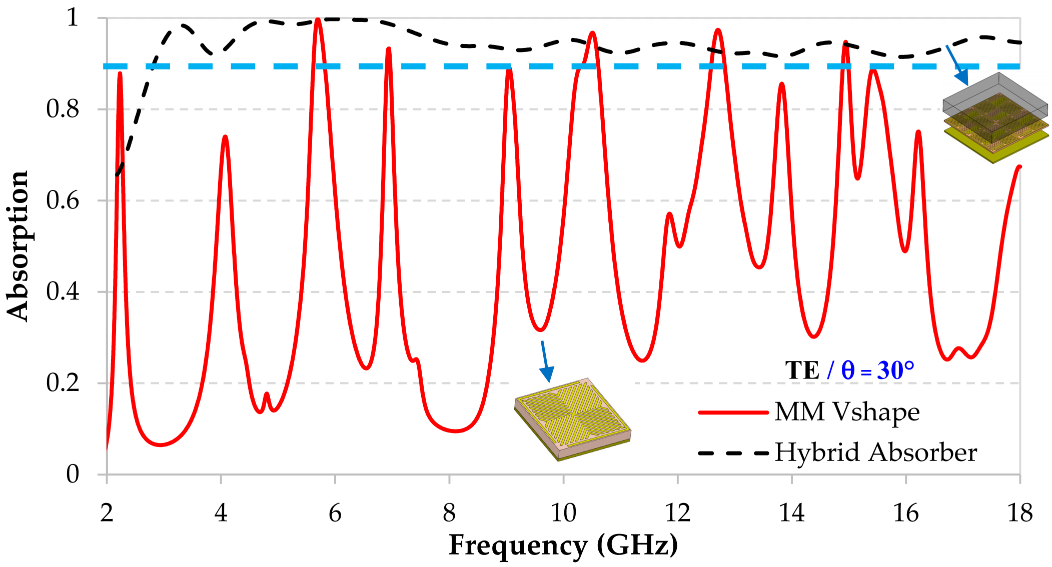

(**a**)

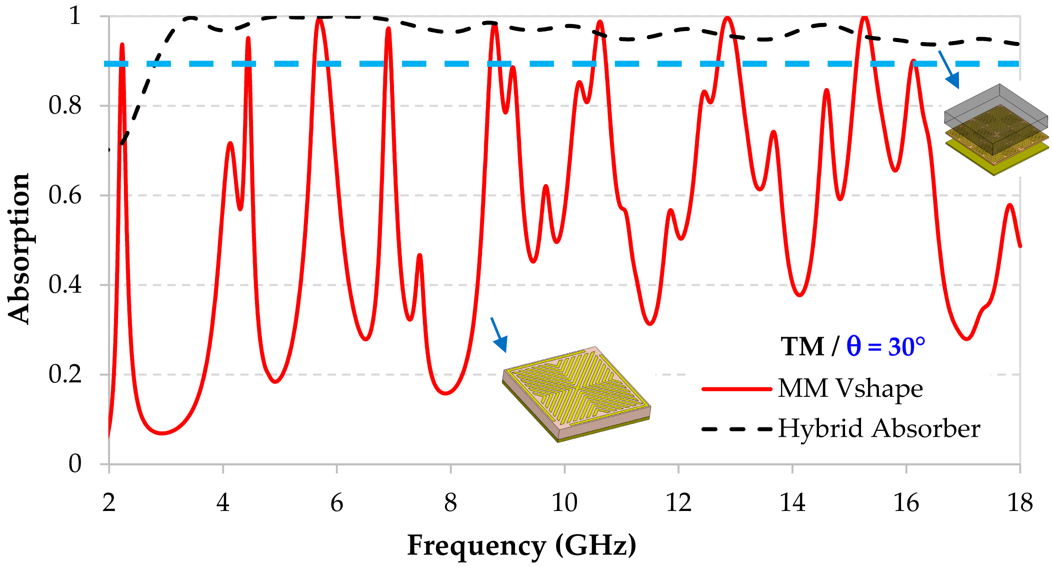

(**b**)

Figure S6. Simulated absorption performance of the hybrid absorber compared to that of the MM at oblique incidence of θ = 30° (**a**) for the TE and (**b**) for the TM polarizations (Dashed blue line presents the absorption value of 90%).

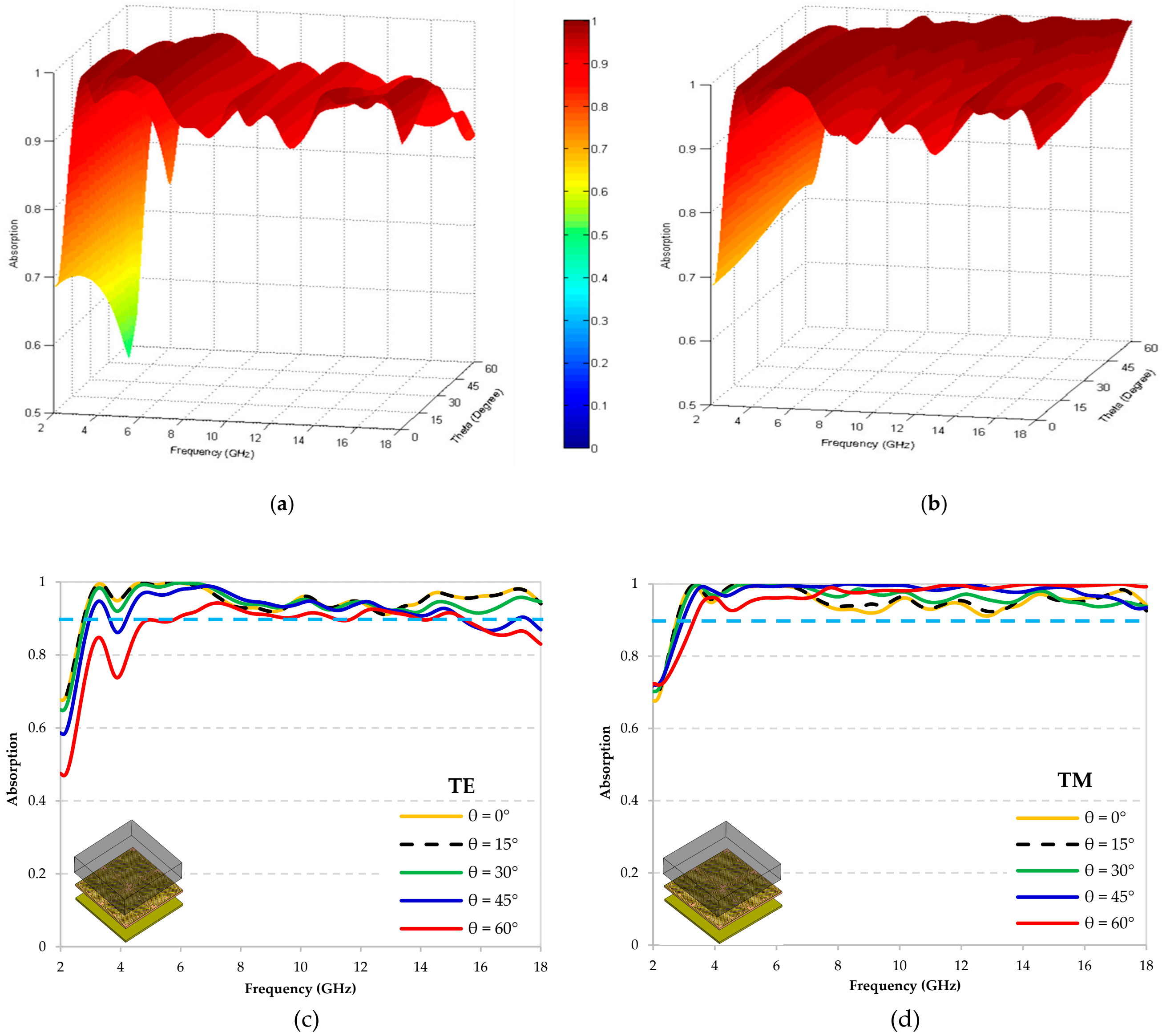

Figure S7. Simulation of the absorption performance of the hybrid absorber with different incidence angles ranging between 0° and 60° for (**a**) TE and (**b**) TM modes (The same scale is used for the two figures); Simulation of the absorption performance of the hybrid absorber with different incidence angles (0°, 15°, 30°, 45° and 60°) for (**c**) TE and (**d**) TM modes (The blue dashed line corresponds to the absorption value of 90%).
